# Supplementary material for: Antibiotic-Induced Immunosuppression—A Focus on Cellular Immunity
Source: Antibiotics (Basel). 2024 Nov 1;13(11):1034. doi: 10.3390/antibiotics13111034 (PMC11591424; doi:10.3390/antibiotics13111034)
Supplement: Supplementary file 1 [file antibiotics-13-01034-s001.zip › antibiotics-3205288-supplementary.pdf]

antibiotics

MDPI

Supplementary Table S1: Summary of antibiotic effects on immune cell function

Immune/ Non-immune cell	Reference/ PMID	Author, Year	Antibiotic	In vivo, in vitro	Animal/ human	Stimulus/ disease	Cell type	Effect	Mechanism
Immune: Basophils	[149]	Abuaf N, 2008	Amoxicillin	In vivo	Human	Allergy	Basophils	Activation - Increased CD203c	-
Immune: Bone marrow	[150]	Leach KL, 2007	Oxazolidinones	In vitro	Human	-	K562 lymphoblast cell line	Mechanistic	Inhibited protein synthesis by crosslinking ribosomal RNA in the peptidyl transfer centre of mitochondrial, but not cytoplasmic, ribosomes
Immune: Dendritic cells	[93]	Lima CMF, 2021	Amoxicillin Penicillin	In vitro	Human	Amoxicillin allergy	Dendritic cells	Cytokine release - Increased IL-6	-
Immune: Dendritic cells & Lymphocytes	[44]	Rodriguez-Pena R, 2006	Amoxicillin	In vivo	Human	Delayed-type hypersensitivity reaction	Dendritic cells T-cells	Antigen presentation– upregulation of HLA-DR & CD86/80	Bidirectional signalling between dendritic cells & T-cells

								Lymphocyte proliferation – increased proliferation, Cytokine release - Decreased IFN	
Immune: Dendritic cells & Lymphocytes	[94]	Juanola O, 2016	Norfloxacin	In vivo	Human Mice	Cirrhosis Bacterial peritonitis	Dendritic cells T-cells	Antigen presentation - Decreased CD80& CD86 Cytokine release - Increased IL-10 Population – Increased Tregs	Treg population changes related to increases in rag1 gene (T-cell receptor processing)
Immune: Eosinophils	[132]	Kohyama T, 1999	Erythromycin Clarithromycin Josamycin Tetracycline Cefazolin	In vitro	Human	Atopy	Eosinophils	Cytokine release – Reduced IL-8 by 14-member macrolides	Effect occurred post-transcriptionally & related to structure of macrolide
Immune: Eosinophils	**11408771**	Cui CH, 2001	Roxithromycin	In vitro	Human	-	Eosinophils	ROS release – Reduced	-
Immune: Eosinophils	10383596	Shoji T, 1999	Roxithromycin	In vivo	Human	Asthma	Peripheral & pulmonary eosinophils	Counts - reduced eosinophils Leukotrienes - reduced	-
Immune: Eosinophils	**10875487**	Amayasu H, 2000	Clarithromycin	In vivo	Human	Asthma	Peripheral & pulmonary eosinophils	Counts - reduced eosinophils Leukotrienes - reduced	-

Immune: Langerhans cells	[53]	Ohshima A, 1998	Roxithromycin	In vitro	Mouse	-	Langerhans cells	Antigen presentation – Reduced HLA-DR expression<br>Cytokine release – Reduced IL- 1β	-
Immune: Lymphocyte	[151]	Lawrence JW, 1996	Ciprofloxacin	In vitro	Mouse	-	L1210 lymphocyte cell line	Cell cytotoxicity	Interfered with mitochondrial topoisomerase II resulting in a loss of mtDNA.
Immune: Lymphocytes	[36]	Koziel R, 2006	Ciprofloxacin	In vitro	Human	-	Jurkat lymphocyte cell line	Cell cytotoxicity	60% reduction of mtDNA content, inhibition of the respiratory chain, & a significant decrease in mitochondrial membrane potential
Immune: Lymphocytes	[48]	Banck G, 1979	Penicillins<br>Cephalosporins<br>Aminoglycosides<br>Chloramphenicol<br>Sulfamethoxazole<br>Trimethoprim<br>Nalidixic acid<br>5-fluorocytosine<br>Erythromycin	In vivo	Human	-	Lymphocytes	Lymphocyte Proliferation – Impaired by erythromycin, clindamycin, & rifampin.<br>No effect with penicillins, cephalosporins,	Protein synthesis inhibited

			Clindamycin Rifampin Fusidic acid Nitrofurantoin Doxycycline					aminoglycosides, chloramphenicol, sulfamethoxazole, trimethoprim, nalidixic acid, & 5-fluorocytosine,	
Immune: Lymphocytes	[56]	Strzepa A, 2016	Enrofloxacin	In vivo	Mouse	Ovalbumin	Lymphocytes	Cytokine release - Production of type-1 (IFN-γ), type-2 (IL-4, IL-5, IL-10, IL-13) & Th17-associated (IL-17A) cytokines was inhibited	-
Immune: Lymphocytes	[59]	Konno S, 1992	Roxithromycin	In vivo	Mouse	Concanavalin A	Lymphocytes	Cytokine release - Initial increase (<14 days) in IL-1/2 but inhibited after 42 days Blastogenesis - Increased	-
Immune: Lymphocytes	[60]	Konno S, 1993	Roxithromycin	In vivo	Mouse	Concanavalin A	Lymphocytes	Cytokine release - Initial increase (<7 days) in IL-1/2 but inhibited after 28 days Blastogenesis – Increased Specific inhibitor of Th2 cells	-

Immune: Lymphocytes	**36891994**	Ghorab MM, 2023	Quinazolinone Benzenesulfonamide	In vivo	Mouse	-	Lymphocytes	Activation - Increased	-
Immune: Lymphocytes	[92]	Park SJ, 2004	Erythromycin	In vivo	Human	Diffuse panbronchiolitis	Pulmonary lymphocytes	Cytokine release – Reduced IL-2 & IFN-γ, increased IL-4, IL-5, IL-13 Shift from Th1 to Th2 phenotype	-
Immune: Lymphocytes & Monocytes	[51]	Pu-lverer G, 199-2	Cefodizime Cefotaxime	In vitro	Mouse	-	Leukocytes Monocytes	Lymphocyte proliferation – Inhibited by Cefotaxime	-
Immune: Lymphocytes & Neutrophils	[106]	Stamatiou R, 2023	Colistin	In vivo	Rat	Emphysema LPS	Neutrophils Lymphocytes	Cytokine release – Reduce IL- 1β, no effect on TNF-α Migration/chemotaxis – Increased Proliferation – No effect Cell death - Increased	Cell death mediated by increased by caspase-3
Immune: Lymphocytes & Neutrophils	[134]	Taw-fik AF, 1919-1	Vancomycin Teicoplanin Daptomycin Coumermycin	In vitro In vivo	Human Mouse	-	Leukocytes Volunteer PBMCs	Phagocytosis - no effect	-
Immune: Lymphocytes & Neutrophils	[122]	Gialdroni Grassi G, 1984	Ceftriaxone	In vitro In vivo	Human	S aureus C albicans	Volunteer neutrophils & lymphocytes	Chemotaxis – Inhibited in vitro Phagocytosis - No effect	-

Immune: Lymphocy tess	[34]	Smith DM, 2002	Lactam 1	In vivo	Huma n	-	Jurkat lymphocyte cell line	Lymphocyte Apoptosis - Increased	Induced DNA damage & inhibited DNA replication. Caused p38 mitogen-activated protein kinase activation, S phase arrest, & apoptotic cell death. p38 was found to be a central player in beta-lactam-induced apoptosis & resided downstream of DNA damage but upstream of caspase activation. Accompanying caspase-8 activation was cleavage of the pro-apoptotic Bcl-2 family protein Bid, & release of the mitochondrial

									cytochrome c. This was also associated with activation of caspase-9 & -3.
Immune: Macrophages	[68]	Yan M, 2017	Danofloxacin	In vivo	Pig	LPS	Alveolar macrophages	Cytokine release - Decreased IL-1β, TNF-α, IL-6, NO (nitric oxide), & PGE2. Increased IL-10	Effects occur pre-translationally
Immune: Macrophages	[146]	Hodge S, 2006	Azithromycin	In vitro	Human	COPD	Alveolar macrophages	Phagocytosis - increased<br>Cytokine release - Deceased	Inhibition of phagocytosis mediated by phosphatidylserine pathway caused inhibition suggesting pathway
Immune: Macrophages	[55]	Liu S, 2023	Doxycycline	In vivo	Mouse	-	Bone marrow derived macrophages	Inflammasome assembly – Inhibited NLRP3<br>Cytokine release - Reduced IL- 1β<br>Apoptosis – Inhibited caspase-1	Inhibited mitochondrial translation
Immune: Macrophages	[101]	Lino Y, 2001	Clarithromycin<br>Roxithromycin	In vivo	Human	Chronic sinusitis	Macrophages	Antigen presentation - increased CD80,	Number of rings in the structure causes effect

								no change in HLA-DR/CD54	
Immune: Macrophages	[105]	Miyata T, 1998	Ampicillin<br>Cephalexin<br>Cefotiam<br>Amikacin<br>Clindamycin<br>Tetracycline<br>Bleomycin	In vitro	Rat	-	Macrophages	Phagocytosis – Reduced by all except ampicillin<br>Chemotaxis - Reduced	-
Immune: Macrophages	[57]	Ogino H, 2009	Ciprofloxacin<br>Gatifloxacin<br>Norfloxacin<br>Levofloxacin	In vivo<br>In vitro	Mouse	LPS	Peritoneal macrophages	Cytokine release - Ciprofloxacin, gatifloxacin, & norfloxacin inhibited both TNF- & IL- 1β production. Levofloxacin inhibited IL- 1β production only. LPS stimulated IL-6 production was inhibited only by norfloxacin.	Greater effect seen with those with cyclopropyl group at the N1 position &/or a piperazinyl group at the C7 position
Immune: Macrophages	[103]	Nunez RM, 1989	Carbopenem	In vitro<br>In vivo	Mouse	C albicans	Peritoneal macrophages	Chemotaxis – Increased<br>Phagocytosis - Increased	-
Immune: Macrophages	[104]	Barriga C, 1996	Teicoplanin<br>Vancomycin	In vivo	Mouse	C albicans	Peritoneal macrophages	Phagocytosis – Enhanced<br>Chemotaxis - Enhanced	-

Immune: Macrophages	[148]	Eswarappa SM, 2008	Folimycin	In vitro	Mouse	LPS	Peritoneal macrophages	Cytokine release – No effect on TNF<br>NO production – Reduced<br>NF-κB - Inhibited	Inhibits V-ATPases, alters intra-Golgi pH, which in turn causes defective processing & reduced surface expression of TLR4<br>NO inhibited pre-translationally, potentially through NF-κB
Immune: Macrophages & Lymphocytes	[54]	Ortega E, 2004	Erythromycin<br>Azithromycin<br>Josamycin	In vivo	Mouse	-	Peritoneal macrophages<br>Splenic lymphocytes	Phagocytosis - Impaired in macrophages<br>Cytokine release – Decreased macrophage IL-12 but increased IL-18 & lymphocyte IL-4	-
Immune: Macrophages & Neutrophils	[140]	Yamaryo T, 2003	Clarithromycin<br>Erythromycin<br>Roxithromycin<br>Oleandomycin<br>Josamycin<br>Spiramycin<br>Clindamicin	In vitro	Human	LPS	Neutrophils<br>Alveolar macrophages	Apoptosis – increased in neutrophils<br>Phagocytosis – Increased in macrophages	Effect only seen in 14-member & 15-member macrolides

			Azithromycin<br>Ampicillin<br>Cefaclor					
Immune: Macrophages & Neutrophils	[58]	Ianaro A, 2000	Roxithromycin<br>Clarithromycin<br>Erythromycin<br>Azithromycin	In vitro<br>In vivo	Rat	Carrageenin pleurisy	Lung neutrophils<br>J774 macrophage cell line	Cytokine release - Decreased prostaglandins & TNF-α	Inhibition of cyclooxygenase-2 & inducible nitric oxide synthase protein expression
Immune: Macrophages & Neutrophils	[33]	Plekhova NG, 2015	Maxifloxacin	In vitro	Mouse	Sterile beef broth<br>S pneumoniae	Peritoneal neutrophils & macrophages	Cell death – Increased<br>ROS production - Increased	Effect reversed by immunomodulation with tinrostim & licopid
Immune: Macrophages & PBMCs	[135]	Mato R, 1992	Lomefloxacin	In vitro	Human<br>Mouse	C albicans	Volunteer PBMCs<br>Peritoneal macrophages	Phagocytosis - no effect	-
Immune: Mast cells	**11001175**	Sugimoto, 2000	Everniomicin<br>Teicoplanin<br>Vancomycin<br>Concanavalin A	In vitro	Rat	-	Peritoneal mast cells	Histamine release – Increased by vancomycin & teicoplanin	-
Immune: Mast cells	10757422	Toyoguchi T, 2000	Vancomycin<br>Miconazole<br>Fluconazole<br>Fosfomycin<br>Cilastin<br>Fluconazole	In vitro	Rat	-	Peritoneal mast cells	Histamine release – Increased by vanc & miconazole	-

Immune: Monocyte & Promyelocyte	[27]	Milosevic TV, 2018	Linezolid Tedizolid	In vitro	Human	-	HL-60 promyelocyte & THP-1 monocyte cell line	Mechanistic	Inhibition of CYTox I expression, cytochrome *c*-oxidase activity, & spare respiratory capacity, causing swelling of the mitochondrial matrix & loss of their cristae
Immune: Monocytes	[82]	Bailly S, 1990a	Ciprofloxacin Pefloxacin Ofloxacin	In vitro	Human	LPS	Volunteer isolated monocytes	Cytokine release - Decreased TNF & IL-1	Impaired protein synthesis rather than impaired release, potentially mediated by quinolone-induced accumulation of intracellular cAMP
Immune: Monocytes	[84]	Khan AA, 1998	Trovafloxacin	In vitro	Human	LPS Heat-killed S aureus	Monocytes	Cytokine synthesis - Reduced IL-1, 6, 10 TNF	-

Immune: Monocytes	[95]	Spyridaki A, 2012	Clarithromycin	In vivo	Human	Ventilator-associated pneumonia & sepsis	Monocytes	Antigen presentation – Decreased CD86 Cytokine release - Decreased TNF, increased IL-10	-
Immune: Monocytes	[69]	Bode C, 2015	Linezolid Vancomycin Daptomycin	In vitro	Human	LPS	THP-1 monocyte cell line	Cytokine release - Linezolid increase IL-1, 6 &10, & TNF. Vancomycin increased IL-6, 10, TNF. Daptomycin increased IL-6/10 but decreased IL-1 TLR expression – Upregulated by linezolid & vancomycin, downregulated daptomycin. Phagocytosis – Increased by vancomycin	Pre-translation effect
Immune: Monocytes	[71]	Ives TJ, 2003	Grepafloxacin	In vitro	Human	S. aureus Zymogen A	THP-1 monocyte cell line	Cytokine release - Reduced IL-1, IL6, IL-8, TNF release ROS production - Reduced	-
Immune: Monocytes	[136]	Muenster S, 2015	Amphotericin Itraconazole Anidulafungin	In vitro	Human	LPS	THP-1 monocyte cell line	Cytokine release - ambisome decreases TNF,	Pre-translation

								itraconazole increases TNF & IL-1, anidulafungin increases IL-1<br>Phagocytosis – Suppressed by ambisome & Itraconazole	
Immune: Monocytes	[15]	Bailly S, 1990b	Ciprofloxacin	In vitro	Human	LPS	Volunteer isolated monocytes	Cytokine release - Decreased monocyte IL-1	Post-transcriptional inhibition
Immune: Monocytes & Neutrophils	[119]	Fietta A, 1986	Teicoplanin<br>Vancomycin	In vitro	Human	S aureus	Volunteer neutrophils & monocytes	Chemotaxis/adherence – No effect<br>Phagocytosis - No effect<br>Killing – Enhanced in monocytes	-
Immune: Monocytes & Neutrophils	[72]	Franks Z, 2013	Linezolid<br>Vancomycin	In vitro<br>In vivo	Human<br>Mouse	MRSA<br>LPS	Volunteer neutrophils & isolated monocytes<br>Mice	Cytokine release - Reduced release of IL- 1β, IL-6 & TNF-α	-
Immune: Monocytes & PBMCs	[70]	Bode C, 2014	Piperacillin<br>Doxycycline<br>Erythromycin<br>Moxifloxacin<br>Gentamicin	In vitro	Human	LPS<br>Cardiac bypass	THP-1 monocyte cell line<br>PBMCs	Cytokine release - Erythromycin, moxifloxacin & doxycycline increased IL- 1β, 6<br>TLR expression - Erythromycin, moxifloxacin &	Pre-translation

								doxycycline increased TLR-1,2,4,6 Phagocytosis – Inhibited by piperacillin, doxycycline & moxifloxacin	
Immune: Neutrophils	[108]	Sugita K, 1995	Ampicillin Methicillin Oxacillin Benicillin Sulbenicillin Ticarcillin Piperacillin Cefotaim Cefoperazone Ceftizoxime Cefmenoxime Ceftazadime Ceftriaxone Cefpimizole Cefuzonam Cefsulodin Cefmetazole Cefbuperazone Latamoxef Flumoxef Erythromycin Josamycin Midekamycin Rokitamycin	In vitro	Human	Volunteers	Neutrophils	Chemotaxis – Inhibited by Minocycline & doxycycline	Chelation of Ca-ions

			Tetracycline<br>Doxycycline<br>Minocycline<br>Gentamicin<br>Tobramycin<br>Amikacin<br>Sisomycin<br>Piromidic acid<br>Cinoxacin<br>Norfloxacin<br>Ofloxacin<br>Enoxacin<br>Ciprofloxacin<br>Rifampicin<br>Chloramphenicol<br>Fosfomycin<br>Lincomycin<br>Clindamycin						
Immune: Neutrophils	[111]	Naess A, 2006	Linezolid	In vitro	Human	Zymosan	Neutrophils	Chemotaxis – No effect<br>Phagocytosis – No effect<br>Respiratory burst – No effect	-
Immune: Neutrophils	[128]	Suzuki H, 1997	Roxithromycin	In vivo	Human	Chronic sinusitis	Neutrophils	Chemotaxis/recruitment - reduced	Impaired IL-8
Immune: Neutrophils	[143]	Herrera-Insua, 1997	Qinupristin<br>Dalfopristin<br>Sparfloxacin	In vitro	Human	E faecium	Neutrophils	Phagocytosis - enhanced	Strain dependant, phagocytosis impaired if vancomycin-

									resistant strain used
Immune: Neutrophils	[147]	Noma T, 1998	Roxithromycin Cefaclor Ofloxacin Aztreonam	In vivo	Human	Seriously handicapped with severe mental retardation	Neutrophils	Phagocytosis – Enhanced by roxithromycin Bactericidal – Enhanced by roxithromycin	-
Immune: Neutrophils	**7759458**	Kamoi H, 1995	Roxithromycin	In vivo	Human	Asthma	Neutrophils	ROS production - reduced	-
Immune: Neutrophils	**36713462**	Pereiro P, 2023	Sulfamethoxazole Clarithromycin	In vivo	Zebra fish & larvae	Carp virus	Neutrophils	Counts - Reduced	Altered transcription of complement components
Immune: Neutrophils	**38147695**	Rieder JC, 2023	Doxycycline	In vitro	Dog	S aureus	Neutrophils	ROS production – Reduced NET release - Increased	-
Immune: Neutrophils	[126]	Sakito O, 1996	Erythromycin Roxiflomycin	In vivo	Human	Diffuse panbronchiolitis	Patient & volunteer neutrophils	Chemotaxis/migration – Reduced Cytokine release – TNF-α & IL- 1β reduced	Impaired IL-8 release
Immune: Neutrophils	[133]	Scaglione F, 1993	Clarithromycin	In vivo	Human	Chronic bronchitis	Patient & volunteer neutrophils	Phagocytosis – enhanced Chemotaxis – no effect	-
Immune: Neutrophils	[125]	Kadota J, 1993	Erythromycin	In vivo	Human Mice	Diffuse panbronchiolitis	Patient, volunteer & mice	Chemotaxis/migration - reduced	Impaired chemotactic gradient (IL-8)

							pulmonary neutrophils		
Immune: Neutrophils	[88]	Banerjee D, 2004	Clarithromycin	In vivo	Human	COPD	Pulmonary neutrophils	Chemotaxis – Reduced Cytokine release - No effect on IL-8/TNF	-
Immune: Neutrophils	[123]	Oda 1994	Erythromycin	In vivo	Human	Diffuse panbronchiolitis	Pulmonary neutrophils	Chemotaxis - Inhibited	Impairs chemokine gradient
Immune: Neutrophils	[124]	Oda H, 1995	Erythromycin	In vivo	Human	Diffuse panbronchiolitis	Pulmonary neutrophils	Chemotaxis/migration - Inhibited	Inhibits chemokine leukotriene B4 production
Immune: Neutrophils	[91]	Simpson JL, 2007	Clarithromycin	In vivo	Human	Asthma	Sputum neutrophils	Counts – Reduced Cytokines – Reduced IL-8	IL-8 mediated drop in numbers
Immune: Neutrophils	[127]	Piacentini GL. 2007	Azithromycin	In vivo	Human	Paediatric asthma	Sputum neutrophils	Count - Reduced	-
Immune: Neutrophils	[139]	Pasqui AL, 1995	Imipenem	In vitro In vivo	Human	Elderly Diabetic	Volunteer & patient neutrophils	Phagocytosis - increased Oxidative burst – Increased	-
Immune: Neutrophils	[138]	Scheffer J, 1992	Cefaclor Cefetamet Ro 40-6890	In vitro	Human	E coli P aeruginosa P mirabilis	Volunteer neutrophils	Phagocytosis – Increased (not Ro) Bactericidal – Enhanced Leukotriene release - Decreased	In class differences in actions

Immune: Neutrophils	[79]	Matera G, 1995	Meropenem	In vitro	Human	PMA<br>LPS	Volunteer neutrophils & monocytes	Phagocytosis - Reduced<br>ROS production - Reduced<br>Chemotaxis - no effect<br>Cytokine release - reduced TNF (but not IL-1/6/8)	-
Immune: Neutrophils	[49]	Kushiya K, 2005	Azithromycin<br>Rokitamycin<br>Vancomycin<br>Teicoplanin<br>Arbekacin<br>Linezolid	In vivo	Human	Toxic shock syndrome toxin-1	Volunteer neutrophils	Cytokine release - Macrolides reduced production, Vancomycin, teicoplanin, linezolid, & arbekacin,, no effect<br>Proliferation – No effect	-
Immune: Neutrophils	[80]	-Reato G, 1-999	Co-amoxiclav	In vitro	Human	-	Volunteer neutrophils	Cytokine release - enhanced IL-8 & IL- 1β release<br>Phagocytosis - enhanced	-
Immune: Neutrophils	[81]	Lankelma JM, 2017	Ciprofloxacin<br>Vancomycin<br>Metronidazole	In vivo	Human	LPS<br>S pneumoniae<br>K pneumonia<br>E coli	Volunteer neutrophils	Cytokine release - no effect<br>Chemotaxis/migration – No effect	-
Immune: Neutrophils	[87]	Yoshimura T, 1996	Levofloxacin	In vitro	Human	PHA	Volunteer neutrophils	Cytokine release - Increased IL-2,	-

								reduced IL- 1β, no effect on IL-8	
Immune: Neutrophils	[107]	Anderson R, 1989	Erythromycin<br>Roxithromycin	In vitro	Human	-	Volunteer neutrophils	Chemotaxis – Increased<br>ROS production - decreased	Enhance neutrophil migration by an antioxidant mechanism that is not due to inhibition of transductional events involved in the activation of NADPH-oxidase or to oxidant scavenging properties
Immune: Neutrophils	[109]	Belsheim JA, 1981	Benzylpenicillin<br>Ampicillin<br>Mecillinam<br>Cefuroxime<br>Cefoxitin<br>Cefotaxime<br>Ceftriaxone<br>Lymecycline<br>Doxycycline<br>Gentamycin<br>Amikacin	In vitro	Human	E coli<br>P aeruginosa	Volunteer neutrophils	Chemotaxis – inhibited by aminoglycosides & tetracycline	-
Immune: Neutrophils	[110]	Fietta A, 1983	Carbenicillin<br>Piperacillin<br>Thienamycin	In vitro	Human	S aureus	Volunteer neutrophils	Chemotaxis – Inhibited by cephalosporins	-

			Cefotetan<br>Ceftazidime<br>Moxalactam						
Immune: Neutrophils	[112]	Ballesta S, 2003	Linezolid	In vitro	Human	S aureus<br>E faecalis	Volunteer neutrophils	Phagocytosis – No effect<br>Chemotaxis – No effect	-
Immune: Neutrophils	[113]	Labro MT, 1986	Cefotaxime<br>Cefodizime	In vitro	Human	S aureus	Volunteer neutrophils	Chemotaxis – no effect<br>ROS production - Increased	In class differences in effect on ROS production
Immune: Neutrophils	[115]	Rodriguez AB, 1993	Cefoxitin	In vitro	Human	C albicans	Volunteer neutrophils	Chemotaxis/adherence – Increased<br>Phagocytosis - Increased	-
Immune: Neutrophils	[116]	Rodriguez AB, 1991	Cefmetazole	In vitro	Human	-	Volunteer neutrophils	Chemotaxis – Increased<br>Phagocytosis – Increased<br>ROS production - Increased	-
Immune: Neutrophils	[117]	Burgaleta C, 1987	Cefotaxime<br>Cefoxitin<br>Ceftazidime<br>Latamoxef<br>Amikacin<br>Sisomicin<br>Tobramycin	In vitro	Human	C albicans	Volunteer neutrophils	Chemotaxis/migration – Impaired by cephalosporins<br>Phagocytosis – No effect	In class effects on migration
Immune: Neutrophils	[118]	Capodicasa E, 1991	Teicoplanin<br>Vancomycin	In vitro	Human	C albicans	Volunteer neutrophils	Chemotaxis/adherence – Inhibited<br>Phagocytosis – Inhibited	High doses only

Immune: Neutrophils	[120]	Moran FJ, 1991	Teicoplanin Vancomycin	In vitro	Human	C albicans	Volunteer neutrophils	Chemotaxis – Inhibited Phagocytosis – no effect	-
Immune: Neutrophils	[121]	Schultz MJ, 2000	Erythromycin	In vivo	Human	S pneumoniae	Volunteer neutrophils	Cytokine release - reduced chemokine (IL-8) production	-
Immune: Neutrophils	[137]	Wenisch C, 1996	Azithromycin Clarithromycin Roxithromycin	In vitro	Human	E coli	Volunteer neutrophils	Phagocytosis – Impaired by azithromycin & clarithromycin ROS production – Decreased by azithromycin	-
Immune: Neutrophils	[141]	-Braga PC, 1-997	Rokitamycin	In vitro	Human	-	Volunteer neutrophils	Phagocytosis – no effect ROS production - reduced	-
Immune: Neutrophils	[142]	Lianou PE, 1993	Ciprofloxacin	In vivo in vitro	Human	-	Volunteer neutrophils	Phagocytosis – enhanced Chemotaxis – no effect Bacterial killing – No effect	-
Immune: Neutrophils	[144]	Forsgren A, 1985	Ciprofloxacin Norfloxacin Ofloxacin	In vitro	Human	Zymosan S aureus Chemotactic peptide	Volunteer neutrophils	Phagocytosis – No effect Killing - Enhanced	Enhanced killing by direct bacterial effect only
Immune: Neutrophils	[145]	Gruger T, 2008	Pipemidic acid Cinoxacin Norfloxacin	In vitro	Human	C albicans	Volunteer neutrophils	Phagocytosis – Inhibited at high dose by	Effect related to structure of fluroquinolone

			Lomefloxacin Enoxacin Ciprofloxacin Ofloxacin Levofloxacin Enrofloxacin Moxifloxacin Gatifloxacin Sparfloxacin Garenoxacin					Ciprofloxacin, Garenoxacin, Moxifloxacin, Enoxacin Oxidative burst – Inhibited at high dose by Ciprofloxacin, Garenoxacin, Moxifloxacin Activation – Increased CD11b expression at high dose by Ciprofloxacin, Garenoxacin, Moxifloxacin Killing – Increased at high dose by norfloxacin & sparfloxacin	s with effects seen in those with a cyclopropyl- moiety at position N1 only
Immune: Neutrophils	8560094	Mitsuyama T, 1995	Erythromycin	In vitro	Human	fMLP PMA	Volunteer neutrophils	Neutrophil ROS - Decreased	Cyclic AMP-dependent protein kinase (PKA), H-89 dependant
Immune: Neutrophils	[75]	Foca A, 1993	Teicoplanin	In vitro	Human	LPS	Volunteer neutrophils	Cytokine release - reduced IL- 1β, IL-8 TNF	-

Immune: Neutrophils	[76]	Schultz MJ, 1998	Erythromycin<br>Penicillin	In vitro	Human	Heat-killed S pneumoniae	Volunteer neutrophils	Cytokine release - Erythromycin decreased TNF & IL-6, & IL-10, IL-12 & IFN-γ at high dose	Il-6 inhibition was mediated by TNF inhibition
Immune: Neutrophils & PBMCs	[114]	Fietta A, 1994	Cefixime<br>Cefdinir	In vitro	Human	Zymosan	Volunteer neutrophils & PBMCs	Phagocytosis – Enhanced by cefdinir<br>Chemotaxis – no effect<br>ROS production – no effect	-
Immune: PBMCs	[45]	Roche Y, 1988	Ciprofloxacin<br>Ofloxacin<br>Pefloxacin	In vivo	Human	Phytohemagglutinin (PHA)	PBMCs	Proliferation – Decreased<br>Cytokine release – Increased IL-2<br>IL-2R – No change	Independent of DNA synthesis
Immune: PBMCs	[46]	Roche Y, 1987	Ciprofloxacin<br>Ofloxacin<br>Pefloxacin	In vivo	Human	Phytohemagglutinin (PHA)	PBMCs	Proliferation – decreased<br>Cytokine release - IL-1 decreased	-
Immune: PBMCs	[47]	Mori S, 2010	Ciprofloxacin	In vivo	Human	Glyceraldehyde-derived AGE	PBMCs	Lymphocyte proliferation – Inhibited<br>Monocyte adhesion – reduced expression<br>Cytokine release - Reduced TNF/IFN	Enhance COX-2 expression increasing cAMP

Immune: PBMCs	[102]	Karakike E, 2022	Clarithromycin	In vivo	Human	Sepsis ARDS	PBMCs	Antigen presentation - upregulated monocyte HLA-DR	Upregulation in genes involved in cholesterol homeostasis
Immune: PBMCs	[73]	Garcia-Roca P, 2006	Erythromycin Linezolid	In vitro	Human	LPS	Volunteer PBMCs	Cytokine release-Reduced monocyte IL-1β, TNF-α & IL-6	-
Immune: PBMCs	[74]	Stevens DL, 1995	Clindamycin Penicillin	In vitro	Human	LPS	Volunteer PBMCs	Cytokine release - clindamycin reduced TNF	Inhibits protein synthesis
Immune: PBMCs	[77]	Vickers IE, 2006	Ciprofloxacin Ceftazidime Cotrimoxazole Piperacillin-tazobactam	In vitro	Human	Heat-killed S maltophila	Volunteer PBMCs	Cytokine release - Co-trimoxazole inhibited TNF secretion at all doses, ciprofloxacin & ceftazidime inhibited at high dose.	-
Immune: PBMCs	[78]	Pichereau S, 2012	Vancomycin Trimethoprim/sulfamethoxazole Tigecycline Daptomycin Linezolid Clindamycin Azithromycin	In vitro	Human	S. aureus toxic shock syndrome toxin-1 (TSST-1) Staphylococcal enterotoxin A (SEA) α-toxin Panton-Valentine	Volunteer PBMCs	Cytokine release - Decreased IL-6 & IFNy by tigecycline, decreased TNF-α & IL-8 by linezolid, increased IL-8 by trimethoprim. IL-1β, IL-6, IL-8, IFN-γ & TNF-α	-

						leucocidin (PVL)		decreased by all antibiotics at v high concentration (>25mg/ml)	
Immune: PBMCs	[83]	Riesbeck K, 1990	Ciprofloxacin	In vitro	Human	-	Volunteer PBMCs	Cytokine release - increased lymph IL-2 but no lymph IFN-γ or monocyte IL- 1β & TNF-α	-
Immune: PBMCs	[85]	Ono Y, 2000	Grepafloxacin	In vitro	Human	-	Volunteer PBMCs	Cytokine release - Reduced IL-1, 6, 8, TNF	Occurs at the transcriptional level
Immune: PBMCs	[100]	Roche Y, 1987	Pefloxacin Ciprofloxacin	In vitro	Human	LPS PHA	Volunteer PBMCs	Antigen presentation – No effect Cytokine release - decreased monocyte IL-1 Proliferation - Decreased	-
Immune: Spleen cells	[98]	Asano K, 2001	Roxithromycin	In vitro	Mouse	Haemocyanin absorbed to aluminium hydroxide	Spleen cells	Antigen presentation - Supressed CD80 & CD866	-
Immune: Spleen cells	[97]	Suzuki M, 2002	Roxithromycin	In vitro In vivo	Mouse	Haemocyanin absorbed to aluminium hydroxide	Splenic B-cells	Antigen presentation – Suppressed CD86 & CD80 (but only after 4weeks)	-

Immune: Spleen cells	[50]	Karrow NA, 2001	Clarithromycin	In vivo	Mouse	-	Splenic macrophages, NK & lymphocytes	Proliferation - no effect	-
Immune: Spleen cells	**28957452**	Cheng RY, 2017	Vancomycin<br>Ceftriaxone	In vitro	Mouse	-	Splenic Treg cells	Differentiation - ceftriaxone decreased splenic Tregs	Modulated via gut microbiome
Immune: Spleen cells	[99]	Kawazu K, 2000	Roxithromycin	In vivo	Mouse	Ovabumin	Splenocytes	Antigen presentation - no effect on CD80/86	-
Immune: T-cells	[43]	Schmid DA 2006	Ciprofloxacin<br>Norfloxacin<br>Moxifloxacin	In vitro	Human	Delayed hypersensitivity reactions	T-cells	Proliferation – Increased by all	Cross reactivity with t-cell receptor causing direct stimulation
Immune: T-cells	[86]	Kaminshi MM, 2010	Ciprofloxacin	In vitro	Human	Atopic dermatitis	T-cells	Cytokine release – Reduced IL-2 & IL-4<br>ROS production - Reduced	Caused a loss of mtDNA & decreased activity of complex 1. Leads to reduction in NF-κB & AP-1 transcription factors
Mixed: Immune: Bone marrow & Lymphocyte	**[52]**	Neftel KA, 1986	Amoxicillin<br>Azthreonam<br>6-Aminopenicillanic acid	In vitro<br>In vivo	Human<br>Chickens<br>Mouse	Orthopaedic surgery	Bone marrow cells<br>Chick embryo liver cells<br>Mouse lymphoma	Lymphocyte proliferation – All inhibited in a dose dependant fashion	-

Non-immune: Chick embryo	7-Desacetoxycephalosporanic acid<br>Carbenicillin<br>Ticarcillin<br>Piperacillin<br>Methicillin<br>Penicillin-G<br>Mezlocillin<br>Azlocillin<br>Cloxacillin<br>Oxacillin<br>Flucloxacillin<br>7-Desacetylcephalosporanic acid<br>Ceftriaxone<br>Cefoxitin<br>Moxalactam<br>7-Cephalosporanic acid<br>Cefmenoxime<br>Ceftizoxime<br>N-formimidoyl-Thienamycin<br>Cephalothin<br>Clavulanic acid<br>Ceftazidime<br>Cefazolin<br>Cephalexin<br>Cefuroxime<br>Cefotaxime<br>Cefotiam	YAC-1 & EL4 cell lines

Mixed: Immune: Bone marrow Non-immune: Renal & ovarian	[152]	Nagiec EE, 2005	Eperezolid	In vitro	Human Hamster	-	K562 erythroleukemia cells, HEK renal, & CHO ovarian cell lines	Cell proliferation - inhibited	Decrease in mitochondrial cytochrome oxidase subunit I levels, consistent with an inhibition of mitochondrial protein synthesis.
Mixed: Immune: PBMCs Non-immune: Skin nerve fibres	[31]	Garrabou G, 2017	Linezolid	In vitro	Human	Joint infections	PBMCs Skin nerve fibres	Mitochondrial-dependant apoptosis - Increased	Reduced mitochondrial protein levels, complex IV activity, & mitochondrial mass. Certain mitochondrial polymorphisms more susceptible
Mixed: Immune: Bone marrow Non-immune: Cardiac, Hepatic, Renal	[26]	McKee EE, - 2006	Chloramphenicol Tetracycline Erythromycin Azithromycin Clindamycin Kasugamycin Lincomycin Streptomycin Eperezolid Linezolid	In vitro	Rat Rabbit	-	Isolated heart, liver, & bone marrow mitochondria	Mitochondrial toxicity - Oxazolidinones chloramphenicol & tetracycline inhibit mitochondrial protein synthesis. Macrolides, lincosamides, &	-

								aminoglycosides no effect	
Non-immune: Bladder	[38]	Aranha O, 2002	Ciprofloxacin	In vitro	Human	-	HTB9 bladder cell line	Mitochondrial Induced apoptosis - Increased	Mitochondrial depolarisation disruption of calcium homeostasis, cytochrome C release, caspase-3 activation, mitochondrial swelling & Bcl-2 dependant redistribution of Bax to the mitochondrial membrane
Non-immune: Brain	[66]	Mike JK, 2023	Azithromycin	In vivo	Sheep	Hypoxic-encephalopathy	Brain tissue	Cytokine release - Reduced il-6	-
Non-immune: Breast	[35]	Chen D, 2008	Lactam 1	In vivo	Mouse	-	Breast cancer cell line	Apoptosis - Increased	induction of DNA damage leading to apoptosis
Non-immune: Breast	[39]	Yu M, 2016	Levofloxacin	In vivo	Human	-	Breast cancer cell line	Proliferation – Inhibited Apoptosis - Increased s	Deactivation of PI3K/Akt/mTOR & MAPK/ERK pathways

Non-immune: Cochlear	[29]	Desa DE, 2018	Gentamicin	In vitro	Mouse	-	Isolated cochlear explants	Mitochondrial dysfunction - Increased	The rapid conversion of highly reactive O2.- to H2O2 occurs during the acute stage of ototoxic antibiotic exposure & the endogenous antioxidant system is significantly altered.
Non-immune: Colon & Hepatic	[37]	Herold C, 2002	Ciprofloxacin	In vitro	Huma n	-	CC-531, SW403, HT-29 colon & HepG2 cell lines	Mitochondrial Induced apoptosis - Increased<br>Cell proliferation - Reduced	Suppressed mtDNA synthesis, increased upregulation of Bax & of the activity of caspases 3, 8 & 9, & decreased mitochondrial membrane potential
Non-immune: Enzyme	[153]	Morris JC, 1996	Gentamicin<br>Kanamycin A<br>G418	Organochem istry	*Bacillu s cereus*	-	Isolated phosphatidylin ositol phospholipase	Mechanistic	Act as allosteric activators of phospholipase c

Non-immune: Hepatic, Muscle, Renal	[32]	De Vriese AS, 2006	Linezolid	In vivo	Human Rat	Linezolid induced optic neuropathy, encephalopathy, skeletal myopathy, lactic acidosis, & renal failure	Muscle, liver, & kidney tissue	Mechanistic	Inhibits mitochondrial protein synthesis with no effect on mtDNA
Non-immune: Histology & Serum	**29406285**	Takahashi E, 2017	Clarithromycin	In vivo	Mouse	Influenza A	Serum Lung histology	Migration/chemotaxis – Reduced Cytokine release – No effect on IL-6, MCP-1, IFN-γ, TNF-α, MIP-1α	--
Non-immune: Lung	[40]	Song M, 2016	Levofloxacin	In vitro	Human	-	A549, H3255, NCL-69 & H460 lung cell lines	Proliferation - Inhibited Mitochondrial-dependant apoptosis - Increased	Inhibits activities of mitochondrial electron transport chain complex I & III, leading to inhibition of mitochondrial respiration & reduction of ATP production
Non-immune: Mechanistic	[20]	Hong S, 2015	Apramycin Gentamicin Kanamycin A Hygromycin B	Organochemistry	Human	-	Isolated mitochondrial 23S rRNA	Mechanistic	Direct binding of aminoglycosides to helix 69 of human

									ribosomal RNA
Non-immune: Multiple: Renal & Hepatic	[21]	O'Reilly M, 2019	Gentamicin	In vitro	Rat	-	Isolated renal cortical & hepatic cell mitochondria	Mitochondrial dysfunction - Increased	Gentamicin behaves as an uncoupler of the electron transport chain (ETC) Stimulates State 4 & inhibits State 3u mitochondrial respiration leading to collapse of mitochondrial membrane potential & reduced ROS production
Non-immune: Multiple: Renal & Hepatic	[22]	Simmons CF, 1980	Gentamicin	In vitro	Rat	-	Isolated renal cortical & hepatic cell mitochondria	Mitochondrial dysfunction - Increased	Inhibits Stage 3 mitochondrial respiration. Drop in whole kidney ATP concentration
Non-immune: Multiple: Renal & Hepatic	[24]	Weinberg JM, 1980, b	Gentamicin Neomycin Kanamycin Streptomycin	In vitro	Rat	-	Isolated renal cortical & hepatic cell mitochondria	Mitochondrial dysfunction - Increase	Stimulates State 4 mitochondrial respiration & inhibits State 3

									& DNP-uncoupled respiration The potency of the aminoglycosides in producing these effects strongly correlated with the number of ionizable amino groups present on the aminoglycoside molecule suggesting that cationic charge is an important molecular determinant of aminoglycoside-induced mitochondrial toxicity.
Non-immune: Multiple: Renal & Hepatic	[25]	Yang CL, 1995	Gentamicin	In vitro	Rat	-	Isolated renal cortical cell mitochondria	Mitochondrial dysfunction - Increased	Enhanced superoxide anion & hydroxyl radical generation

Non-immune: Renal	[19]	Weinberg JM, 1980, a	Gentamicin	In vitro	Rat	-	Isolated renal cortical cell mitochondria	Mitochondrial dysfunction - Increased	Increased mitochondrial Stage 4 respiration. Enhanced uptake of sodium- & potassium-acetate enhancing energy-dependant swelling
Non-immune: Renal	[23]	Ueda N, 1993	Gentamicin	In vitro	Rat	-	Isolated renal cortical cell mitochondria	Mitochondrial dysfunction - Increased	Increased hydrogen peroxidase production mobilised mitochondrial iron release
Non-Immune: Renal	[41]	Denamur S, 2016	Gentamicin	In vitro	Pig	-	LLC-PK1 renal cell line	Mitochondrial-dependant apoptosis - Increased	ROS dependant increase in p53 levels resulted in accumulation of p21 & of phospho-eIF2α. These effects could be related to an impairment of

									proteasome as we demonstrated an inhibition of trypsin- & caspase-like activities. Moderate endoplasmic reticulum stress could also participate to cellular toxicity induced by gentamicin, with activation of caspase-12 without change in GRP74 & GRP98.
Non-Immune: Renal	[42]	Servais H, 2005	Gentamicin	In vitro	Pig	-	LLC-PK1 renal cell line	Mitochondrial Induced apoptosis -Increased	Within 2 h, gentamicin induced a partial relocalisation [from lysosomes to cytosol] of the weak organic base acridine

									orange followed by a loss of mitochondrial membrane potential, release of cytochrome c from granules to cytosol, & the activation of caspase-9 (as from 12 h, & increase in caspase-3
Non-immune: Renal	[28]	Morales AI, 2010	Gentamicin	In vivo In vitro	Rat	Metformin	Renal cortical cells	Mitochondrial dysfunction - Increased	Gentamicin depleted respiratory components (cytochrome c, NADH), probably due to the opening of mitochondrial transition pores & increased reactive oxygen species production from the

									electron transfer chain.
Non-immune: Respiratory fluid	[61]	Breslow-Deckman JM, 2013	Linezolid	In vivo	Mouse	Influenza then S. pneumonia	BAL fluid	Cytokine release - Decreased IFN-γ & TNF-α	-
Non-immune: Respiratory fluid	[62]	Jacqueline C, 2014	Linezolid Vancomycin	In vivo	Mouse	MRSA	BAL fluid	Cytokine release – Linezolid decreased IL- 1β MIP2 & TNF-α	-
Non-immune: Respiratory fluid	[63]	Kaku N, 2016	Tedizolid Linezolid Vancomycin	In vivo	Mouse	MRSA	BAL fluid	Cytokine release – Linezolid & tedizolid decreased TNF-α, IL-6 & MIP-2,	-
Non-immune: Respiratory fluid	[64]	Yanagihara K, 2009	Linezolid	In vivo	Mouse	MRSA	BAL fluid	Cytokine release - Decreased	-
Non-immune: Respiratory fluid	[65]	Verma AK, 2019	Linezolid	In vivo	Mouse	Influenza then MRSA	BAL fluid	Cytokine release - Decreased	-
Non-Immune: Respiratory fluid	[129]	Cervin A, 2008	Clarithromycin	In vitro	Human	Chronic rhinosinusitis	Nasal lavage	Cytokine release – Reduced IL-8	-
Non-Immune: Respiratory fluid	[130]	Wallwork B, 2006	Roxithromycin	In vivo	Human	Chronic sinusitis	Nasal lavage	Cytokine release – Reduced IL-8	-

Non-Immune: Respiratory fluid	[131]	Yamada T, 2000	Clarithromycin	In vivo	Human	Chronic sinusitis	Nasal lavage	Cytokine release – reduced IL-8	-
Non-Immune: Respiratory fluid	[90]	Fonseca-Aten M, 2006	Clarithromycin	In vivo	Human	Paediatric asthma	Nasaopharyngeal aspirates	Cytokine release - Reduced TNF-α, IL- 1β, IL-10	-
Non-immune: Respiratory fluid & Serum	[67]	Luna CM, 2009	Linezolid	In vivo	Pig	MRSA	Serum & BAL cytokines	Cytokine release – No effect	-
Non-immune: Respiratory Fluid & Serum	[89]	Cameron EJ, 2013	Azithromycin	In vivo	Human	Smokers with asthma	Sputum aspirates & serum	Cytokine release – No effect	-
Non-immune: Serum	**26917573**	Van Opstal E, 2016	Vancomycin	In vivo	Mouse	C. difficile	Serum	Humoral immunity - Reduced antibodies (IgG/M)	-
Non-immune: Skeletal	**26657404**	Protti A, 2016	Linezolid	In vitro	Human	Linezolid-induced lactic acidosis	Skeletal muscle	Lactic acidosis	Diminished global oxygen consumption & extraction reflective of selective inhibition of mitochondrial protein

								synthesis (probably translation) with secondary mitonuclear imbalance.
Non-immune: Zebra fish larvae	36427668	Liu S, 2023	Chlortetracycline Oxytetracycline	In vivo	Zebra fish larvae	-	Larvae	Increased NF-κB regulated gene expression
